# Do small hydrogen-containing gas molecules support low deuterium in mitochondrial water?

**DOI:** 10.3389/fmicb.2026.1933444

**Published:** 2026-10-09

**Authors:** Stephanie Seneff

**Affiliations:** Computer Science and Artificial Intelligence Laboratory, Massachusetts Institute of Technology, Cambridge, MA, United States

**Keywords:** ammonia, deuterium, glutamine, hydrogen peroxide, microbiome, mitochondria, molecular hydrogen, NADPH oxidase

## Abstract

Deuterium is a natural heavy isotope of hydrogen, containing a neutron as well as a proton, making it twice as heavy. Mitochondrial ATP synthase nanomotors are highly sensitive to deuterium, because it impedes the flow of protons through the nanomotors and causes the γ C-terminal helix to unwind. It appears that metabolic processes have evolved to devise ways to reduce the deuterium load in the mitochondria, primarily, we argue, by exploiting several small hydrogen-containing molecular gases. We propose that, when a gas is produced, deuterium, due to its extra weight, tends to stay behind in the aqueous medium, so the gas becomes deuterium-depleted. Furthermore, many of the enzymes that synthesize and/or metabolize these small gas molecules fractionate out deuterium by exploiting proton tunneling. We hypothesize that the gut microbes play a crucial role in producing deuterium-depleted gas molecules, such as hydrogen gas (H_2_), methane (CH_4_), ammonia (NH_3_), hydrogen sulfide (H_2_S), trimethylamine (TMA), formaldehyde (HCHO), and methanethiol (CH_3_SH). Gut microbial metabolism of TMA likely provides a low-deuterium source of one-carbon (1C) units to fuel the methylation pathway in the host. The glutamate-glutamine exchange that takes place between astrocytes and neurons plausibly capitalizes on NH_3_ to preferentially deliver ^1^H (protons) to neuronal mitochondria. Red blood cells actively import NH_3_ while the cation NH_4_^+^ is excreted in the urine, possibly as a strategy to fractionate out deuterium. In this paper, we investigate how small molecular hydrogen-containing gases are handled in metabolism, from a perspective that considers that these gases may serve a useful role in reducing the deuterium content in the mitochondria. Our hypothesis awaits validation by expert researchers who can perform appropriate experiments to test the hypothesis.

## 1 Introduction

Deuterium is a heavy isotope of hydrogen, having a neutron as well as a proton in the nucleus, making it essentially twice as heavy. Deuterium is pervasive in nature, found in seawater at a concentration of 156 parts per million (ppm). It is present in water, organic molecules, and the atmosphere, wherever normal hydrogen is found. There is a growing awareness in the research community that deuterium has biological properties that can disrupt metabolism (Répás et al., 2025). In particular, a theoretical paper has argued that the ATP synthase nanomotors in the mitochondria are very sensitive to deuterium, as it causes molecular “stuttering,” mechanical drag, and increased leakage of reactive oxygen species (ROS) (Olgun, 2007). While 156 ppm may seem rare, because hydrogen is so common, deuterium levels in the blood are five or six times as high as calcium levels (Yaglova et al., 2023).

Human metabolic policy appears to have evolved to sustain a sophisticated strategy for maintaining very low deuterium in the mitochondrial intermembrane space, to minimize exposure of the ATPase nanomotors to deuterium. The process likely depends heavily on the gut microbiome to recycle nutrients by producing abundant molecular hydrogen gas and using the hydrogen gas as a reducing agent to create short-chain fatty acids (SCFAs) and methyl groups to fuel the methylation pathway (Seneff et al., 2026). It has been suggested that these microbial products based on recycling hydrogen gas would likely have significantly reduced deuterium content, due to the properties of specialized flavoproteins that exploit proton tunneling, which deuterons are less capable of, and to the fact that deuterium tends to stay behind in the aqueous medium when a gas is formed (Seneff and Kyriakopoulos, 2025a).

A seminal paper published in 1961 showed that molecular hydrogen produced by a marine pseudomonad contained 80% less deuterium than the deuterium fraction in the seawater (Krichevsky et al., 1961). The hydrogenase enzyme expressed by this microbe bears significant homology with hydrogenase enzymes expressed by multiple microbes inhabiting the human gut. It is therefore plausible that the hydrogen gas produced in the gut is similarly depleted in deuterium.

Raindrops collected on the ground in tropical areas are enriched in deuterium compared to raindrops captured in colder climates or on mountain tops. This is due to the fact that rain samples evaporate as they fall through the atmosphere, and the water that leaves the raindrop as water vapor tends to leave deuterium behind in the liquid phase (Peng et al., 2007; Chen et al., 2021). This fact provides evidence of what might be a general principle that deuterium resists entering the gas phase.

Interestingly, methane gas formed at high temperature during volcanic eruptions has also been found to be extremely depleted in deuterium (Ricci et al., 2024). This further supports a pattern that deuterium tends to remain in the aqueous medium, given that deuterium is twice as heavy as hydrogen. Like methane and molecular hydrogen, trimethylamine (TMA) is also a gas at room temperature. This may mean that it is also naturally reduced in deuterium content, since we believe this may be a general property of hydrogen-containing gases. We will have more to say about TMA later in this paper, since gut microbes produce it from dietary sources such as L-carnitine, choline, and betaine. Other hydrogen-containing gases produced by gut microbes include hydrogen sulfide (H_2_S), methanethiol (CH_3_SH), formaldehyde (HCHO), and dimethyl sulfide (CH_3_)_2_S (see Figure 1). Might it be the case that all these small molecular gases are enriched in ^1^H? Furthermore, does human biology exploit this property? This is the topic that this paper addresses.

**Figure 1:**
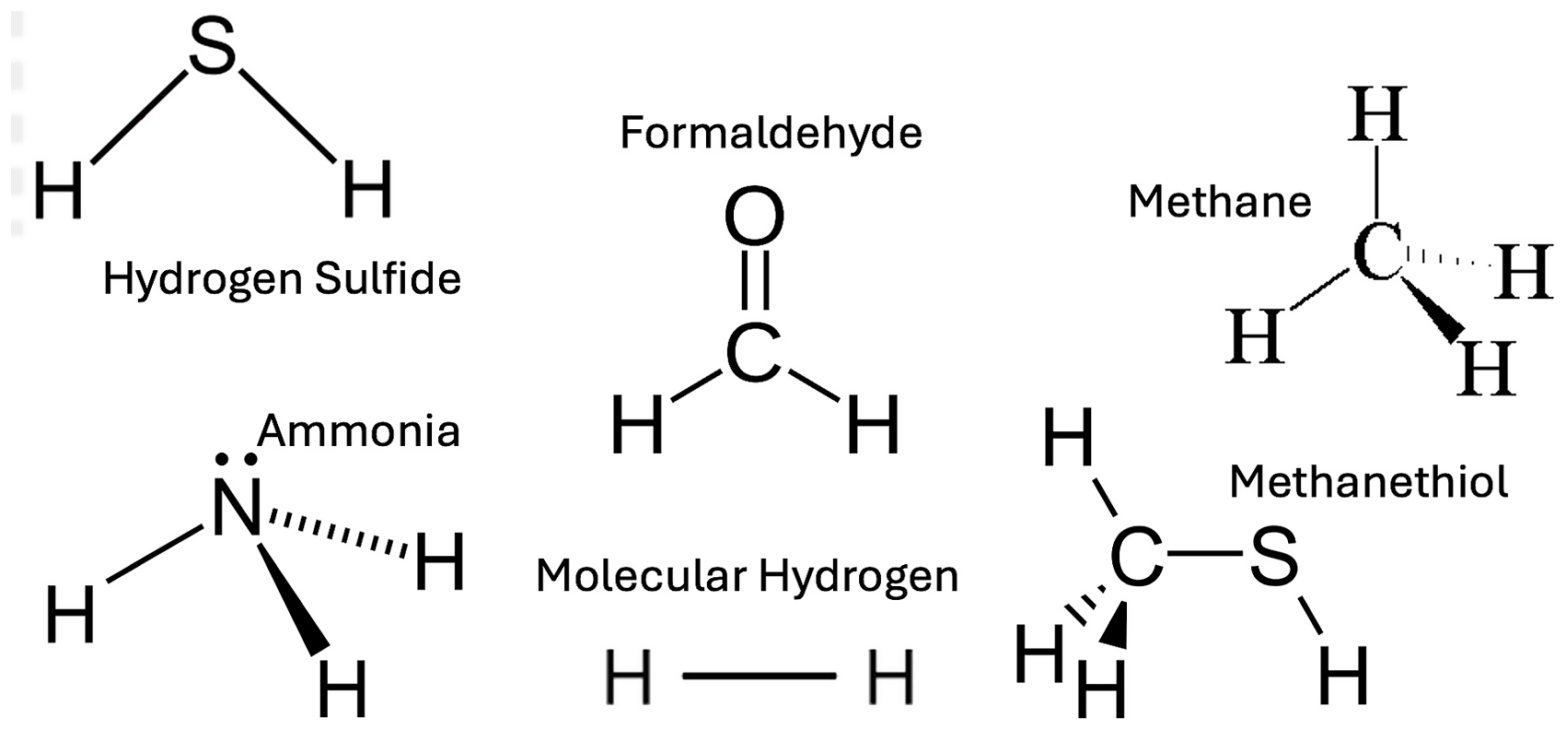
Some small molecular gases produced by gut microbes that contain hydrogen.

Protons bound to carbon, nitrogen, oxygen, and sulfur differ in their ability to freely exchange with deuterons in the medium, and their ability to exchange also depends on the surrounding molecular context. Protons bound to oxygen in carboxyl groups or hydroxyl groups exchange with deuterons from the aqueous medium much more readily than protons bound to nitrogen in amide groups, such as the C(=O)NH_2_ amide group in glutamine (Grocholska and Bąchor, 2021). Glutamine is the most abundant free amino acid in human plasma (Cruzat et al., 2018), and it plays a powerful role in protecting from ammonia toxicity (Nakamura and Hagen, 2002; Bassini-Cameron et al., 2008). We will present an argument in this paper that glutamine’s amide group may be enriched in ^1^H, a feature that may be exploited by human biology.

Generally, carbon atoms are very good at holding onto protons. The protons bound to carbon atoms in organic molecules for the most part remain securely bound and never exchange with deuterons or protons from the aqueous medium, although there are some interesting exceptions such as the protons in bis-allylic carbon atoms and in rings containing carbon and nitrogen, such as the pyrrolidine ring in proline and the imidazole ring in histidine (Seneff et al., 2025).

## 2 Evidence that deuterium damages ATPase nanomotors

F_O_F_1_ ATP synthase (ATPase) converts electrochemical energy from a proton gradient into mechanical rotation, which drives the chemical synthesis of ATP. The F_O_ domain is a membrane-spanning proton channel that acts as a rotary motor driven by the flow of protons down their electrochemical gradient (Elston et al., 1998). The ATPase nanomotors convert the energy of moving protons into chemical energy for the cell with 75 to 83% efficiency [2]. Deuteronation significantly slows down the nanomotors, causing molecular crowding in the TCA cycle (Boros, 2016). Specifically, deuterons severely compromise proton on–off loading onto the Asp61 residue in the C protein, which is the heart of the biological nanomotor (Aksimentiev et al., 2004). The acid dissociation constant for a deuteron from the Asp61 amino acid residue of the F_O_ rapidly rotating inner mitochondrial membrane-bound subunit is only about 1/3 of the acid dissociation constant for a proton (Elston et al., 1998). The yeast plasma membrane vacuolar H^+^-ATPase, which operates in the reverse of the F_O_F_1_ ATPase nanomotors, pumps protons out of the cell, and it strongly resists or entirely fails to utilize deuterium as a functional substitute for protons during active transport (Kotyk et al., 1990). The yeast V-ATPase pumps and the F_O_F_1_-ATPase nanomotors are in the same superfamily, and both utilize a rotary mechanism to perform their functions (Kane and Parra, 2000), although their rotation schemes differ (Imamura et al., 2005), so there is reason to believe that this feature of the ATPase pump may also apply to some degree to the ATPase nanomotors.

Hydrogen-deuterium exchange (HDX) mass spectrometry of *Escherichia coli* (*E. coli*) ATPase showed that rotation of the γ subunit leads to greatly enhanced deuteration specifically in the γ C-terminal helix, due to better access to the water molecules in the medium. Deuterium trapping and resulting unfolding partially stalls the rotor. This creates substantial frictional or rotational resistance, causing the γ C-terminal tip to get stuck and temporarily unfold while the rest of the shaft keeps rotating (Murcia Rios et al., 2018).

Under anoxic crisis, the ATP nanomotors run in reverse mode to produce ATP, in order to prevent a total collapse of the proton motive force (St-Pierre et al., 2000; Domínguez-Zorita and Cuezva, 2023). The γ C-terminal helix becomes destabilized under such conditions, and it shows greatly enhanced hydrogen/deuterium exchange due to enhanced access to the aqueous medium (Vahidi et al., 2016).

## 3 Methanogens, methanotrophs, methylotrophs, and trimethylamine

Gut microbes ferment complex carbohydrates such as dietary fiber into small organic molecules such as glucose and formate that are then metabolized anaerobically to produce hydrogen gas (Campbell et al., 2023). This hydrogen gas can then be used by methanogens (anaerobic archaea) to generate methane gas directly by reduction of carbon dioxide (CO_2_) (Buan, 2018). For an excellent review on the unique metabolic profile of methanogenic archaea, please see Costa and Whitman (2023). Methane oxidizing genes are a common feature of the human gut microbiome. Methanotrophs such as *Methylocystis intestini* help reduce methane content in the gut while producing useful energy in the form of nicotinamide adenine dinucleotide (NADH) (Zhao et al., 2026). Due to its origins, this NADH is likely a good source of ^1^H rather than ^2^H released into metabolic water by the action of NADH dehydrogenase in the mitochondria.

Methylotrophs metabolize methylated amines, most notably, TMA, to produce formaldehyde (derived from the methyl groups) and ammonia as a byproduct. Formaldehyde can then be processed through assimilatory pathways to yield biomass or through dissimilatory pathways, producing carbon dioxide gas and useful energy as NADH (Klein et al., 2022). Methylotrophs significantly outcompete methanogens for hydrogen gas. Methylotrophs have a much lower threshold for hydrogen gas, consuming it at partial pressures as low as 0.1 Pascal, whereas methanogens require a range from 2.8 to 10 Pascals (Feldewert et al., 2020). The activity of methylotrophs is thus primarily limited by the availability of methyl groups. The proton in NADH that they produce is unlikely to be a deuteron.

Together with Professor László Boros, I recently published a paper hypothesizing that hydrogen gas produced by gut microbes plays an essential role in reducing the deuterium load in host mitochondria. Furthermore, we argued that trimethylamine oxide (TMAO) a metabolite produced by the liver through oxidation of TMA, may serve as a marker for deuterium overload in the mitochondria, systemically (Seneff and Boros, 2026). TMA that remains unmetabolized by gut microbes is an indicator of a deficiency in methylotrophs. Methylotrophs likely play a powerful role in supplying deuterium-depleted methyl groups to the methylation pathway. Therefore, elevated TMAO is a potential indicator of deuterium overload in the mitochondria as well as a disrupted microbiome. TMAO has been well recognized as a risk factor for cardiovascular disease as well as several other chronic diseases (Gatarek and Kaluzna-Czaplinska, 2021; Canyelles et al., 2023).

## 4 TMA is a source of one-carbon units for the methylation pathway

The metabolism of TMA by gut microbes likely plays an important role in reducing the deuterium content in one-carbon (1C) units that feed into the methylation pathway. TMA is produced by gut microbes via the breakdown of a number of different dietary sources containing a methylated amines. These include L-carnitine, choline, betaine, dimethylglycine, and even TMAO (found in fish) (Hoyles et al., 2018). A choline-deficient diet in rats produces a 31–40% reduction in liver folate content, 50% lower hepatic S-adenosylmethionine (SAMe) levels, and a doubling of plasma homocysteine (Bortz and Obeid, 2025), all indicators of deficiencies in the methylation pathway.

TMA can be fully metabolized by gut microbes via TMA dehydrogenase (TMADH), dimethylamine dehydrogenase (DMADH), and methylamine dehydrogenase (MADH), ultimately producing three molecules of formaldehyde, also a gas, and one molecule of ammonia. For example, it has been discovered that the genus *Bilophila* possess the gene pool needed to demethylate TMA, thus reducing the production of TMAO by the liver (Kivenson and Giovannoni, 2020). An analysis of twelve different species of methylotrophs confirmed that all of them were able to grow with TMA as sole source of carbon. In fact, they can also grow on dimethylamine and methylamine (Colby and Zatman, 1973). Formaldehyde is highly reactive, but glutathione-dependent formaldehyde dehydrogenase reduces it to formate, a much less reactive molecule (Martínez et al., 2005).

TMADH is a homodimeric iron–sulfur flavoprotein that interacts with a covalently bound FMN cofactor to extract a hydride ion from TMA (Shi et al., 2005). Flavoproteins generally show a strong deuterium KIE, and TMADH is no exception. Both TMADH and MADH have been shown experimentally to have a high deuterium KIE for the extraction of a hydride ion from their substrates. Presumably due to vibrationally assisted hydrogen tunneling during breakage of the substrate CH bond, TMADH highly favors undeuterated TMA over deuterated TMA. The KIE depends strongly on pH, and it ranges from 4.2 at pH 11 to 6.9 at pH 7.5 to 20.4 at pH 6 (Basran et al., 2001). A deuterium KIE of 17.2 was measured for the hydrogen abstraction step in the reaction catalyzed by MADH (Brooks et al., 1993). Even if the original TMA molecule was not deuterium-depleted, the formaldehyde molecules derived from it will be.

A study on amine oxidation by TMADH using dimethylamine as substrate to slow the reaction down confirmed that it is the neutral amine (the gas) that acts as the substrate rather than the ionized form of the molecule. At low pH, the protonated substrate binds, but is then deprotonated by an active-site residue prior to oxidation. This suggests that the enzyme only accepts the gas form as substrate. These authors also determined the deuterium KIE for K_cat_/K_m_ with dimethylamine as substrate, and found that it had a strong dependence on pH, approaching 1.0 at pH 7 but rising to as much as 8.0 at pH 9.5 (Wanninayake et al., 2019).

Formate is metabolized by anaerobic gut microbes into molecular hydrogen and CO_2_ via formate dehydrogenase during the fermentation process (Pinske and Sawers, 2016). Formate produced from formaldehyde sourced from TMA is likely already deuterium-depleted, due to the high deuterium KIEs of the dehydrogenase enzymes that produced formaldehyde, which may be related to the fact that both the substrate and the product are gases. The hydrogen gas can then be used to synthesize methane, acetate, and butyrate, which all become valuable sources of ^1^H for the mitochondria.

The TMA that remains unmetabolized by the gut microbes is likely enriched in deuterium, due to the fact that the dehydrogenases favored non-deuterated TMA as substrate. This TMA is oxidized to TMAO in the liver, and TMAO is elevated in the serum in association with a large number of chronic diseases (Gatarek and Kaluzna-Czaplinska, 2021; Canyelles et al., 2023).

Formate is very useful to the human host as a feeder into the methylation pathway. Formate and THF are combined by a sequence of reactions catalyzed by the versatile human cytosolic enzyme methylene-THF dehydrogenase, cyclohydrolase and formyl-THF synthetase 1 (MTHFD1) to produce methylene-THF, which is then reduced to methyl-THF by MTHFR to support the methylation pathway (Pietzke et al., 2020). Methylene-THF is also essential for the synthesis of purines and thymidylate (MacFarlane et al., 2009). Mitochondrial formyl-THF synthetase (MTHFD1L) plays an essential role during embryonic development to support the flow of mitochondrial 1C units into the methylation pathway (Pike et al., 2010).

## 5 Experimental evidence that deuterated precursors increase plasma TMAO levels

A paper published in 2011 involved a study where mice were administered synthetic phosphatidylcholine as a supplement, where all the protons in the methyl groups were replaced with deuterons (D9-PC). They recovered D9-TMAO from the plasma, and, furthermore, extended the study to include deuterated choline, deuterated TMAO, and deuterated betaine. All of these supplements resulted in the upregulation of multiple scavenger receptors linked to atherosclerosis (Wang et al., 2011). A mouse study in which the mice were fed deuterated L-carnitine also showed a sharp increase in plasma TMAO following supplementation, further supporting the idea that deuteration is the primary factor promoting TMAO accumulation (Koeth et al., 2013).

A very informative study involving human subjects was conducted by Wilcox et al. (2021). These authors compared natural choline intake from dietary sources such as eggs with supplemental synthetic choline bitartrate. They found that the synthetic supplement sharply raised blood TMAO levels, but participants consuming four eggs daily showed no significant increase in plasma TMAO. In sharp contrast to the effects of synthetic supplements, a survey involving over 14,000 participants found that dietary choline protects from both heart disease and stroke (Zhou et al., 2023). These studies all suggest that the natural forms of these molecules are very low in deuterium, and that this may be their primary beneficial effect.

It appears feasible to design a controlled study to specifically quantify differences in the plasma levels of TMAO when mice are administered natural, undeuterated synthetic, or fully deuterated L-choline and/or L-carnitine. It is anticipated that TMAO levels would rise most in the context of the fully deuterated supplement, and perhaps not at all in the case of the natural forms. With the deuterated supplement, researchers could also track the degree to which the deuterium is recovered in the faeces or in the urine. Our hypothesis predicts that very little of the deuterium should end up in the faeces, because deuterated TMA would be passed on to the liver and eventually excreted in the urine as TMAO. It should also be feasible to simply measure the amount of deuterium in choline sourced from eggs and other foods that are naturally enriched in choline. Such an experiment would likely provide a definitive answer as to whether choline’s nitrogen atoms carry very little deuterium in their methyl groups.

## 6 Do hydrogenases produce low-deuterium molecular hydrogen?

Hydrogenase enzymes are found in thousands of genomes from over 30 different phyla (Vignais and Billoud, 2007; Greening et al., 2016). They are common in aquatic, terrestrial, and even host-associated ecosystems. They are all metalloenzymes that use iron and nickel as catalysts, with [NiFe]-hydrogenases, [FeFe]-hydrogenases and, less commonly, [Fe]-hydrogenases all having been identified. Their reversible reaction catalyzes the oxidation of molecular hydrogen (H_2_) into 2H^+^ and 2e^−^. It has been hypothesized that, during prehistoric times on Earth, the atmosphere was rich in hydrogen gas, which has been proposed to have been the first electron donor leading to ATP synthesis. H_2_ is ubiquitous throughout the biosphere, and it readily diffuses through microbial cells and has a low activation energy requirement. H_2_ metabolizing microbes are keystone species driving carbon cycling along oxygen gradients in hydrogen-rich soil ecosystems (Pich-Choquette and Constant, 2019).

The reaction catalyzed by hydrogenases is reversible, and our interest is in the use of this enzyme to produce molecular hydrogen from protons and electrons by gut microbes. As documented in a paper published in 1961, MI Krichevsky et al. studied a hydrogenase produced by a marine pseudomonad species and found that it was capable of fractionating hydrogen isotopes to a remarkable degree when producing hydrogen gas. The hydrogen gas it produced was greatly depleted in deuterium compared to ocean water, having a deuterium content that was reduced by a factor of five. This microbe produced molecular hydrogen when it was grown with glucose, pyruvate, or formate as nutrients (Krichevsky et al., 1961). While the species investigated was a marine microbe rather than a colonizer of the human gut, its hydrogenase closely resembles hydrogenases synthesized by gut microbes. Furthermore, other studies have shown that hydrogenase enzymes can have extremely high deuterium kinetic isotope effects (KIEs), as high as 43 in an acidic environment (Greene et al., 2015). Part of the reason for this may be the biophysical property that deuterium, being twice as heavy, tends to stay behind in solution in the water and resists the gas phase.

## 7 Microbial production and utilization of hydrogen gas

Molecular hydrogen is increasingly being recognized as a therapy that has widespread benefits. Yıldız et al., in a review paper published in 2025, wrote in the abstract: “H_2_ demonstrates numerous biologically therapeutic properties, including anti-inflammatory, antioxidant, anti-cancer, anti-stress, anti-apoptotic, anti-allergic effects, signaling molecule functions, regulation of redox balance, modulation of antioxidant enzyme gene expression, improvement of blood vessel function, down-regulation of pro-inflammatory cytokines, stimulation of energy metabolism, and protection of the nervous system” (Yıldız et al., 2025).

A metagenomics survey revealed that *Firmicutes* and *Bacteroides* are the most prevalent hydrogen-producing bacteria in the human large intestine, accounting for 92% of the bacterial species (Wolf et al., 2016; Ichikawa et al., 2023). These species break down dietary fiber into small molecules and then extract hydrogen atoms from those molecules. Interestingly, *Bacteroides fragilis*, an important symbiont, anchor themselves in the colonic mucosa, mediated by immunoglobulin A, where they constantly produce hydrogen gas and also protect from pathogens (Donaldson et al., 2018; Ichikawa et al., 2023). Every day, as many as ten liters of hydrogen gas are produced in the human intestines (Levitt, 1969). A study based in Japan showed that patients with dementia had a reduced *Bacteroides* population compared to controls (Saji et al., 2019). Hydrogen gas is currently being studied as an experimental therapy for dementia (Ono et al., 2023).

Seventy percent of the microbial species in the gut have the capacity to metabolize H_2_. The three major pathways for disposal of H_2_ in the gut are methanogenesis, acetogenesis and sulfate reduction, with methanogenic archaea being the most abundant in the healthy gut (Nava et al., 2012). Hydrogen gas that is not captured by the microbes freely enters the blood-stream, and it easily penetrates the blood–brain barrier, where it may serve a role in protecting from oxidative stress (Ichikawa et al., 2023).

Anaerobic metabolism of non-digestible fiber by human gut microbes converts complex carbohydrates into carbon dioxide and hydrogen gas, that, as we have argued, is likely severely depleted in deuterium. Methanogenic archaea then use the hydrogen gas to reduce carbon dioxide to methane gas. This prevents acidification of the gut and facilitates the breakdown of complex carbohydrates, fibers, and resistant starches, increasing the total energy harvest from food (Chen et al., 2024). There is a reduced prevalence of methanogenic archaea in inflammatory bowel disease (IBD) (Cisek et al., 2024). *Methanobrevibacter smithii* is the dominant archaeon in the human gut, accounting for over 90% of the archaeal species in the gut (Volmer et al., 2023). If, as we argue, the hydrogen gas has lost 80% of the deuterium present in the aquatic medium, then the methane gas will also be significantly depleted in deuterium.

Methane is further processed in the gut in order to capture the gas as a significant nutrient. After monooxygenase converts methane to methanol, two subsequent steps involving microbial dehydrogenases transform methanol into formaldehyde and, finally, formate, where NAD^+^ picks up ^1^H to become NADH (which is then potentially a source of ^1^H to fuel the ATPase nanomotors).


methane→methanol→formaldehyde→formate

Formate is widespread as a respiratory energy source in natural habitats (Sawers, 2025). *Campylobacter jejuni*, a common cause of diarrhea, has a periplasmic formate dehydrogenase enzyme which allows formate oxidation to be coupled with microaerobic oxygen reduction to water (Kassem et al., 2017). The water is released in the periplasmic space rather than the cytoplasm, and the outer membrane of the bacterium is highly permeable to water. This plausibly means that the reaction delivers deuterium-depleted water to the host’s gut lumen.

In addition to its conversion to formate via a dehydrogenase, formaldehyde can also be a source of methyl groups in the methylation pathways. A study investigating metabolism of a methylotrophic bacterium, *Methylobacterium extorquens*, when fed methanol as a fuel source, revealed that methanol was reduced to formaldehyde which was then utilized to synthesize methylene tetrahydrofolate from tetrahydrofolate, either through a direct nonenzymatic transfer of the methylene group from formaldehyde to tetrahydrofolate, or through an indirect metabolic pathway involving several steps (Marx et al., 2005). Formaldehyde is highly reactive, and, by being directed down these pathways, it can be quickly metabolized prior to causing harm through other spontaneous reactions. However, a further implication is that the methyl groups in methyltetrahydrofolate will likely be enriched in ^1^H. The fact that methylation pathways are so important in biology may be connected to their ability to deliver ^1^H to mitochondria (Seneff and Boros, 2026; Seneff et al., 2026).

Acetogenic bacteria also use the hydrogen gas to produce acetate from carbon dioxide, according to the Wood-Ljungdahl pathway:


$${\mathrm{2CO}}_{2}+{\mathrm{4H}}_{2}\to {\mathrm{CH}}_{3}{\mathrm{COO}}^{-}+H^{+}+{\mathrm{2H}}_{2}O$$

Both the acetate and the water that are produced in this way are likely to be enriched in ^1^H. Acetate is a precursor to the four-carbon fatty acid, butyrate, the primary nutrient consumed by the colonocytes lining the gut barrier (Hodgkinson et al., 2023). Butyrate stimulates human enteroendocrine L-cells to release glucagon-like peptide-1 (GLP-1), the hormone that suppresses appetite to maintain an ideal body weight (Yadav et al., 2013). GLP-1 analogues (receptor agonists) are widely prescribed to help people lose weight (Ahmad et al., 2026). Butyrate’s beneficial effects in the gut may be primarily due to its low deuterium content.

Finally, hydrogen gas is also used by sulfate-reducing bacteria such as *Desulfovibrio* sp. to reduce sulfate to sulfide according to the following equation (Rowland et al., 2018):


$${\mathrm{4H}}_{2}+S{O_{4}}^{2}+H^{+}\to {\mathrm{HS}}^{-}+{\mathrm{4H}}_{2}O$$

The four water molecules are likely enriched in ^1^H compared to ^2^H. Mitochondria aggressively take up H_2_S (a gas) and oxidize it through the enzyme sulfide quinone oxidoreductase (SQR), which donates electrons to the electron transport chain (ETC) to produce ATP, while releasing (preferentially ^1^H?) protons into the mitochondrial water (Paul et al., 2021). We will return to a discussion on H_2_S in a later section.

We predict that these products of molecular hydrogen metabolism - acetate, butyrate, methane and its derivatives, including formaldehyde, formate, and the methyl groups carried by the methylation pathways, as well as the water produced in reactions involving oxygen reduction, will all be sources of ^1^H that can be used to fuel mitochondria in the human host cells.

## 8 Methane gas: a broader view

Many toxic metal and chemical exposures can alter the composition of the gut microbiome, including pesticides, toxic metals, persistent organic pollutants, and artificial sweeteners (Claus et al., 2017). Gut dysbiosis results in a decrease in beneficial microbiota, such as F*aecalibacterium* and *Roseburia*, along with an increase in pathogenic species, such as adherent-invasive *E. coli* (AIEC). This results in a reduction in SCFAs along with an increase in detrimental microbial metabolites (Kou et al., 2025). Pathogen overgrowth triggers the immune system to release ROS to neutralize the threat, but this can also be harmful to host cells (Novaes et al., 2019).

Excessive methane in the breath, linked to constipation issues, is considered to be a marker for gut dysbiosis, whereby the metabolism of methane gas cannot keep up with its production by methanogenic archaea (Mehravar et al., 2025). However, since 2012, there has been growing interest in the idea of the potential for methane gas administration to have therapeutic benefits. Beneficial effects have been shown in treating ischemia and reperfusion injury, as well as inflammatory diseases in general. It appears to have anti-inflammatory effects and to protect cells from apoptosis (Jia et al., 2018; Ye et al., 2020). This could be simply because it may be a low-deuterium nutrient.

While it had long been believed that methane is primarily synthesized by methanogenic archaea, which reduce carbon dioxide using hydrogen gas as a reducing agent, it has recently become clear that methane can also be generated nonenzymatically from certain common compounds containing methyl groups bound to sulfur or nitrogen atoms. This abiotic mechanism, catalyzed by light and heat, occurs in all living organisms, including cyanobacteria, algae, fungi, plants, and mammals, and it can even take place outside of living cells (Ernst et al., 2023).

Most notably, dimethyl sulfoxide (DMSO) and methionine can be spontaneously oxidized to produce methane under conditions of oxidative stress. Keppler et al. wrote in 2023: “In summary, the reaction of methylated sulfur compounds, such as DMSO and methionine with Fenton-type chemistry involving ROS, carbonate radicals, or oxo-iron(IV) results in the formation of methyl radicals (•CH_3_), of which a portion reacts to CH_4_ through abstraction of a hydrogen atom from hydrocarbons, hydrogen peroxide, or hydrogen carbonate” (Keppler et al., 2023). In an earlier paper by the same authors, it was stated that “An in-depth understanding of the various factors that control cellular methane formation and consumption and a thorough understanding of its dual function and bioactivity may bring significant benefits to the domain of human health and disease” (Keppler et al., 2022).

It is intriguing that, under conditions of oxidative stress, which develops in response to pathogen overgrowth, methane can be regenerated nonenzymatically from methylated nitrogen or sulfur sourced from common organic molecules, via the Fenton reaction. This may be especially important because oxidative stress interferes with the enzymatic production of methane by strict anaerobes (Lyu et al., 2018).

Deuterium loading can sometimes greatly alter the properties of organic molecules. A fascinating study on a fully deuterated triethylammonium cation in a protic ionic liquid, triethylammonium methanesulfonate, revealed remarkably strong kinetic isotope effects via deuterium solid-state NMR spectroscopy. Simultaneously present static and mobile states indicate plastic crystal behavior in this molecule. Full deuteration of the ethyl groups in the trialkylammonium cations tremendously shifts the equilibrium towards the static state. This was associated with a significantly reduced standard molar transition entropy (Khudozhitkov et al., 2022). The larger significance of this phenomenon related to other molecules is unknown, but it is striking that the components of this molecule - the methane, the ethyl groups, the ammonia, and the sulfonate, are all prominent molecules involved in microbial metabolism.

## 9 Hydrogen sulfide gas

Hydrogen sulfide (H_2_S) has long been recognized as a toxic and dangerous gas, but it was much more recently that it also became recognized as an endogenous regulatory transmitter. H_2_S, produced by several human enzymes, affects a variety of biological processes, including transcriptional and posttranslational gene modification, activation of signaling pathways, and alterations in metabolic processes. New efforts are being considered around the use of H_2_S as a gas transmitter with therapeutic drug potential (Jin et al., 2020).

### 9.1 Sulfate reducing bacteria in the gut

*Desulfovibrio* (DSV) spp. are resident commensal bacteria present in the human gastrointestinal (GI) tract, and the most common sulfur-reducing bacteria. They are known to be opportunistic pathogens that frequently overgrow under dysbiotic conditions, e.g., in association with IBD (Singh et al., 2023) and neurodegenerative diseases such as Parkinson’s disease (PD) (Murros et al., 2021). They are a gram-negative bacterium that produces H_2_S via the dissimilatory sulfate reduction pathway, using molecular hydrogen as a reducing agent (Barton et al., 2014). The nematode *Caenorhabditis elegans* exhibits dietary preferences, often favoring bacterial strains that support its growth and health (Shtonda and Avery, 2006). In an elegant study involving a transgenic strain of *C. elegans* which expresses human α-synuclein, researchers found that, when presented with DSV strains from various sources as food, *C. elegans* greatly preferred the environmental strain *D. vulgaris DSM 644* over two human isolates, one of which was obtained from a PD patient. Furthermore, colonization with the environmental strain led to reduced aggregation of α-synuclein, reduced oxidative stress, and increased lifespan (by a median of 36 days). The authors proposed that sublethal stressors may activate protective molecular pathways such as antioxidant defenses that increase the capacity of the host to cope with toxic exposures that could cause cellular damage (Mohammadi et al., 2025).

*Desulfovibrio* strains are generally considered to be strict anaerobes. However, interestingly, it has been found that a strain of *Desulfovibrio* (*D. vulgaris Hildenborough*) obtained from anoxic ditch sludge could tolerate up to 0.04% oxygen in the medium, which could even support their growth. When exposed to an oxygen gradient, these microbes are drawn to an optimal location where the oxygen levels are favorable to growth, i.e., a hypoxic but not an anoxic environment (Johnson et al., 1997). This suggests that they colonize the gut wall where the environment is hypoxic, and they may serve a useful role in clearing the oxygen present in the gut wall, where there is a strong oxygen gradient, benefiting other strict anaerobes colonizing the gut. Singhal et al. noted in a paper published in 2020 that the richly vascularized subepithelial mucosa harbor significantly more oxygen than the anaerobic gut lumen, especially in the more distal, colonic regions of the GI tract (Singhal and Shah, 2020). These authors wrote: “The pO_2_ drops precipitously along the radial axis from the intestinal submucosa to the lumen, which is home to trillions of anaerobic microbes” (Singhal and Shah, 2020).

*D. vulgaris Hildenborough* possess membrane-bound terminal oxygen reductases (members of the respiratory cytochrome bd family) that serve as an oxygen scavenger (Borisov et al., 2011; Ramel et al., 2013). They also express soluble rubredoxin oxidases, which reduce oxygen to water, coupling NADH oxidation to oxygen consumption. Oxygen exposure increases their resistance to killing by H_2_O_2_ (Wildschut et al., 2012). Bacterioferritin plays a crucial role in protecting *D. vulgaris Hildenborough* from oxygen toxicity, by sequestering iron. Its gene expression increases dramatically upon oxygen exposure. It also protects both the host cells and other microbes from oxidative stress due to the Fenton reaction (Figueiredo et al., 2012).

### 9.2 *Mitochondria metabolize H*_2_ *S*

Sulfate-reducing bacteria, common in shallow marine sediments and hydrothermal vents, utilize sulfate as the terminal electron acceptor rather than oxygen, and generate H_2_S as the main respiratory by-product. The endosymbiotic hypothesis of mitochondrial origin proposes that mitochondria originate from the integration of an endosymbiotic bacterium into the host cell, a primordial Asgard archaeon (Roger et al., 2017). This may be why eukaryotic mitochondria are able to oxidize H_2_S efficiently to generate ATP. A mitochondrial sulfide oxidation pathway rapidly eliminates H_2_S by oxidizing it to sulfite and thiosulfate. Sulfide quinone oxidoreductase (SQR) and persulfide dioxidase, operating in tandem, yield sulfite from H_2_S. One proton is picked up by the quinone in the inner membrane and the other is released into the aqueous medium. Both would potentially be deuterium-depleted, given that H_2_S is a gas, derived from molecular hydrogen produced by anaerobic hydrogenogens. Glutathione is involved as an intermediary to temporarily form S-sulfanylglutathione (GSSH). Sulfite is then oxidized to sulfate by sulfite oxidase or to thiosulfate by rhodanese. The electrons that are released by SQR are transferred to the ETC at complex III, promoting ATP synthesis (Murphy et al., 2019).

### 9.3 H_2_S is a powerful signaling gas

Occupational exposure to high levels of H_2_S is toxic, because H_2_S blocks the respiratory chain by inhibiting cytochrome c oxidase. However, H_2_S is an endogenous signaling gas, acting as a cytoprotectant and promoting cardiovascular homeostasis and health (Polhemus and Lefer, 2014). It is produced in human cells primarily by three key enzymes: cystathionine β-synthase (CBS), Cystathionine γ-lyase (CSE), and 3-mercaptopyruvate sulfurtransferase (3-MST), using the sulfur-containing amino acids, L-cysteine and homocysteine as substrates (Pan et al., 2012).

The administration of physiological or pharmacological levels of H_2_S attenuates myocardial injury, protects blood vessels, limits inflammation, and regulates blood pressure (Polhemus and Lefer, 2014). Like NO, H_2_S is a potent vasodilator. In mice, genetic deletion of CSE markedly reduces H_2_S levels in the serum, heart, aorta, and other tissues (Yang et al., 2008). H_2_S plays a role in wound healing, and endogenously produced H_2_S mediates angiogenesis induced by vascular endothelial growth factor (VEGF) (Papapetropoulos et al., 2009). In *in vitro* studies, exogenously administered H_2_S at physiologically relevant concentrations induced angiogenesis and stimulated endothelial cell proliferation and migration (Cai et al., 2007; Papapetropoulos et al., 2009).

## 10 Methanethiol, SELENBP1 and cancer

Hydrogen sulfide, methanethiol, and dimethyl sulfide are all volatile sulfur compounds (VSCs) characterized by their low odor thresholds, significant role in halitosis (bad breath), and toxicity at high concentrations. They act as crucial biological mediators and signaling molecules (gasotransmitters) in mammals, influencing blood pressure, inflammation, and neuronal signaling (Tangerman, 2009). Methanethiol (CH_3_SH) is a toxic foul-smelling gas that plays interesting but poorly characterized roles in human physiology. It can be derived from methionine or through methylation of H_2_S. It is overproduced by cancer cells and often found in excess amounts in tumor microenvironments, associated with accelerated disease progression (Philipp et al., 2023b).

Selenium-binding protein 1 (SELENBP1) is a methanethiol oxidase, and it is a critical enzyme for metabolizing methanethiol (Philipp et al., 2021). It catalyzes the conversion of methanethiol to H_2_S, H_2_O_2_, and formaldehyde.


$$H_{2}O+{\mathrm{CH}}_{3}\mathrm{SH}+O_{2}\to H_{2}\mathrm{CO}+H_{2}O_{2}+H_{2}S$$

It depends on both selenium and copper as catalysts (Philipp et al., 2023a). It plays a protective role in ulcerative colitis (Fonseca-Camarillo et al., 2024). Both mouse and human SELENBP1 are abundantly expressed in the liver, lungs, prostate, colon, and pancreas (Elhodaky and Diamond, 2018; Zhang and He, 2022).

Deficiencies in SELENBP1 cause high levels of methanethiol, leading to a “cabbage-like” breath odor known as extraoral halitosis (Pol et al., 2018). SELENBP1 is widely recognized as a tumor suppressor, and it is often downregulated in various epithelial cancers, including lung, colorectal, ovarian, liver, and prostate cancers (Zhang and He, 2022).

Human methyltransferase-like protein 7B (METTL7B; also known as TMT1B) catalyzes the transfer of a methyl group from SAMe to H_2_S, yielding methanethiol, and to other exogenous thiol-containing small molecules (Maldonato et al., 2021). METTL7B is consistently upregulated in various cancers, acting as a promoter of tumor progression and a marker of poor prognosis (Denford and Wilhelm, 2023). In a study on lung adenocarcinoma cells, high expression levels were linked to enhanced cancer cell proliferation, migration, and invasion, and increased resistance to therapies (Li et al., 2022).

Both glucose and methionine are enriched in cancer cells, and the nonenzymatic Maillard reaction between these two metabolites yields methanethiol. Gut microbes also break down methionine via γ-methionine-lyase, to produce methanethiol. Methanethiol-producing strains such as *Fusobacterium nucleatum* are highly abundant in the gut microbiome of colorectal carcinoma (CRC) patients (Philipp et al., 2023b). Colorectal carcinoma (CRC) and hepatocellular carcinoma (HCC) cells produce and release high levels of methanethiol (CH_3_SH) into the tumor microenvironment (TME) due to their dysregulated sulfur metabolism. Elevated levels of methionine, along with reduced expression of SELENBP1 and increased expression of METTL7B, lead to high endogenous production of this volatile compound, which serves as a scent-based marker for cancer (Philipp et al., 2023b).

We hypothesize that the methanethiol that is produced in abundance in association with colon cancer can be exploited by normal cells expressing SELENBP1 in the gut to produce H_2_S, and formaldehyde, both of which are likely readily available sources of deuterium-depleted water to support mitochondrial health. This hypothesis aligns with the general theory we have developed that cancer cells hoard deuterium and supply the resident immune cells with deuterium-depleted resources (Seneff and Kyriakopoulos, 2025a; Seneff and Kyriakopoulos, 2025c; Kyriakopoulos and Seneff, 2026). Indeed, it has been confirmed experimentally that SELENBP1 expression levels are significantly lower in colorectal cancer cells than in adjacent healthy tissue cells (Zhang et al., 2022). Given that it is a gas, it is likely that, like the other small organic gas molecules, methanethiol has reduced levels of deuterium.

Figure 2 illustrates the pathways by which methanethiol is produced, through both microbial and human enzymes, and the pathway by which it is broken down into potentially valuable deuterium-depleted molecules by SELENBP1.

**Figure 2:**
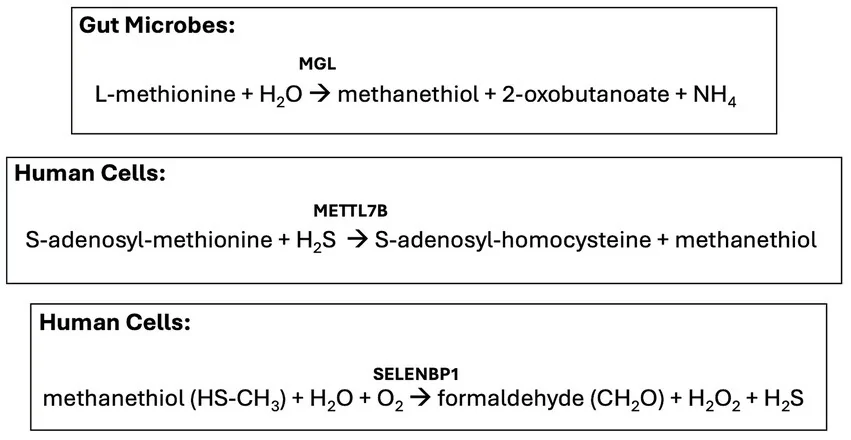
The chemical reactions that involve the production and metabolism of methanethiol. Methanethiol is a major breakdown product of methionine, catalyzed by gut microbes. Human cells can produce methanethiol by methylating hydrogen sulfide gas. Methanethiol is degraded to formaldehyde, hydrogen sulfide, and hydrogen peroxide by SELENBP1. These three metabolites are all likely to be enriched in ^1^H compared to ^2^H. MGL: methionine-γ-lyase; METTL7B: methyltransferase-like protein 7B; SELENBP1: methanethiol oxidase.

## 11 A role for ammonia in deuterium depletion?

Ammonia management in the body is a critical detoxification process centered on converting ammonia, which can be toxic in elevated amounts, into non-toxic urea in the liver (the urea cycle) to be recycled via the gut microbiome or finally for renal excretion. The kidneys play a dual role: managing acid–base balance by excreting ammonia as ammonium (NH_4_^+^) in the urine and producing ammonia from glutamine in the proximal tubules to regulate body pH.

In the remainder of this paper, we will refer to “total” ammonia as T-ammonia, representing the mixture of NH_3_ and NH_4_^+^. NH_3_, being a gas, is likely enriched in ^1^H relative to ^2^H. A question we address here is whether the organism exploits this feature to fractionate deuterium, preferentially retaining NH_3_ and exporting NH_4_^+^.

T-ammonia is no longer viewed merely as metabolic waste. It is a critical driver of tumor progression. T-ammonia accumulates in the tumor microenvironment, where it is a source of nitrogen to support amino acid and nucleic acid synthesis. It stimulates growth pathways and fuels an immunosuppressive state (Chen et al., 2025). Cancer cells actively export ammonia as a gas (NH_3_) which is likely low in deuterium simply because it is a gas. Resident cancer-associated fibroblasts take up NH_3_ and utilize it to synthesize glutamine, which is then delivered back to the tumor cells (Hinow et al., 2021). This exchange may serve as a way to preferentially export ^1^H over ^2^H to the external milieu, by taking advantage of the high pH in the tumor cytoplasm to promote the production and export of NH_3_ derived from T-ammonia (Seneff and Kyriakopoulos, 2025c).

### 11.1 Do gut microbes convert urea to deuterium-depleted ammonia?

Urea, produced primarily in the liver and excreted as a major component of urine, can be viewed as a storage form of T-ammonia. A reaction of urea with one molecule of water yields ammonia and carbon dioxide:


$${\left({\mathrm{NH}}_{2}\right)}_{2}\mathrm{CO}+H_{2}O\to {\mathrm{2NH}}_{3}+{\mathrm{CO}}_{2}$$

The metabolism of urea by microbial urease (Shen et al., 2015), as well as the microbial metabolism of other nitrogen-containing molecules such as the amino acids, purines, and pyrimidines, are all sources of T-ammonia in the gut lumen. Interestingly, ammonia, the gas, (NH_3_) readily diffuses across plasma membranes, whereas ammonium, the ion (NH_4_^+^) does not. Furthermore, epithelial cells in the gut express transport proteins that actively take up NH_3_ but not NH_4_^+^ (Handlogten et al., 2005). Villous epithelial cells throughout the gut express two proteins that facilitate the active transport of NH_3_ across the gut barrier into the circulation, namely the NH_3_ transporter family members Rh B glycoprotein (RhBG) and Rh C glycoprotein (RhCG) (Handlogten et al., 2005).

T-ammonia in the lumen is mainly found in solution as the cation NH_4_^+^. At equilibrium, only a small percentage is present as the gas, NH_3_, particularly in an acidic environment, because of the high acid dissociation constant (pKa). But only NH_3_ can passively cross the gut barrier. Various transport proteins exist to actively carry either NH_3_ or NH_4_^+^ across membranes, and in different circumstances a preference for transport of one or the other is probably strategically important.

It is likely that an ammonium ion containing deuterium (NH_3_D^+^) would leave behind in the lumen the deuteron rather than one of the protons when it gets converted to the gas, ammonia, via acid dissociation: NH_3_D^+^ → NH_3_ + D^+^. This means that ammonia as a gas is likely a source of low-deuterium protons. As the ammonia is syphoned off by the transporters, more ammonia is produced, as the two species restore chemical equilibrium.

### 11.2 What is the role of RhAG in red blood cells?

RhAG, another member of the Rh glycoprotein ammonia transporter family, is only expressed in red blood cells (RBCs). It has not been easy for researchers to figure out what role this protein plays in RBCs, but it is becoming clear that it is involved in T-ammonia homeostasis in the vasculature (Weiner and Verlander, 2010). RhAG acts as a scaffold for the Rh complex. It appears to transport T-ammonia exclusively as NH_3_ across the RBC membrane (Ripoche et al., 2004). It has been suggested that RBCs act as a storage depot for T-ammonia to reduce the levels of free T-ammonia in the circulation (Weiner and Verlander, 2010). Thus, RhAG’s role in RBCs may be to support T-ammonia buffering within the RBCs, which can help sequester T-ammonia and protect the plasma from hyperammonemia (Sudnitsyna et al., 2016).

Notably, RhAG transports methylamine (CH_3_NH_2_) as well as ammonia (NH_3_). Like NH_3_, CH_3_NH_2_ is a gas, and this suggests that RhAG specifically acts as a gas channel. Ripoche et al. wrote: “The results show that RhAG facilitates CH_3_NH_2_/NH_3_ movement across the RBC membrane and represents a potential example of a gas channel in mammalian cells” (Ripoche et al., 2004). In fact, sequestration of methylamine may be a beneficial role of RhAG. The concentration of methylamine in RBCs is five times that found in plasma. Interestingly, methylamine is not efficiently cleared during dialysis treatment, due to the fact that RBCs sequester it (Ponda et al., 2010). An analysis of the crystallographic structure of the related bacterial NH_3_ transport channel AmtB showed, through reconstitution into vesicles, that AmtB conducts uncharged NH_3_ (Khademi et al., 2004), consistent with the analysis of Ripoche et al. for RhAG.

The concentration of T-ammonia in RBCs is significantly higher than that in the plasma. RhAG expressed in RBCs forms a structural unit with anion exchanger 1 (eAE1; a sodium-independent anion exchanger) within the ankyrin core complex. RBCs swell and lyse in isotonic NH_4_^+^ buffer. This effect is tightly regulated by chloride concentration. When chloride was replaced by glutamate, NH_4_^+^-induced swelling was inhibited, preventing hemolysis. Inhibition of eAE1 also completely inhibited hemolysis (Sudnitsyna et al., 2024). It has been shown that NH_3_ transport parallels RhAG expression in RBCs (Ripoche et al., 2004).

Westhoff et al. wrote: “Erythrocytes are ideally positioned to then transport ammonium to be exchanged in the liver and kidney, where other Rh-related proteins (RhBG and RhCG) are expressed” (Westhoff et al., 2004). We take this idea one step further: we suggest that RBCs actively import NH_3_ as a gas from the medullary interstitium in the kidneys, syphoning it off before NH_4_^+^ is exported via the urine, as explained in the next section.

### 11.3 Renal tubules preferentially export NH_4_^+^

The ammonium ion (NH_4_^+^) and potassium ion (K^+^) show almost identical biophysical characteristics. Since NH_4_^+^ is very similar in size and charge to K^+^, cells can exploit K^+^ transport channels to actively transport NH_4_^+^ across membranes. The kidneys utilize a complex process to selectively export NH_4_^+^ into the urine and retain NH_3_ in the interstitium. Cells in the proximal tubules of the kidneys synthesize abundant NH_4_^+^ from glutamine, which is secreted into the luminal fluid through active transport by both an apical Na^+^/H^+^ exchanger NHE3 and by various K^+^ transport proteins (Weiner and Hamm, 2007). However, a substantial amount of ammonia (NH_3_) is reabsorbed later in the thick ascending limb of the loop of Henle. Approximately 40% of the NH_4_^+^ that enters the loop of Henle is passed to the interstitium as NH_3_. As is described below, RBCs siphon off NH_3_ from the interstial pool, and NH_4_^+^ is excreted in the urine.

Tubule cells in the thick ascending limb of Henle have a remarkable apical impermeability to NH_3_, due to the specific lipid composition of the bilayer (Kikeri et al., 1989; Watts and Good, 1994). However, they actively import NH_4_^+^ from the lumen against a concentration gradient, utilizing the Na^+^/H^+^ ion exchanger NHE4, substituting NH_4_^+^ for the proton. Defective versions of NHE4 lead to metabolic acidosis (Houillier and Bourgeois, 2012). On the basolateral side, these cells release NH_3_ but not NH_4_^+^ into the medullary interstitial space, to facilitate acid–base balance and to create a concentration gradient used for final waste excretion (Bourgeois and Houillier, 2024). The Rhesus glycoproteins, RhBG and RhCG act as NH_3_ transporters in the distal tubule and collecting duct (Weiner and Verlander, 2011). We hypothesize that RBCs may actively import NH_3_ in the medullary interstitium, siphoning it off to capture ^1^H for return to the circulation. As we have discussed, red blood cells actively take up NH_3_ but not NH_4_^+^ via their RhAG proteins (Ripoche et al., 2004).

The Inner Medullary Collecting Duct (IMCD), located in the deep, inner medulla, is the final, terminal segment of the renal nephron responsible for maximal urine concentration and final electrolyte adjustment before urine enters the renal pelvis. In the IMCD, basolateral Na^+^-K^+^ ATPase enables active basolateral uptake of NH_4_^+^ but not NH_3_ (Weiner and Verlander, 2011). The renal excretion of NH_4_^+^ acidifies the urine, playing a crucial role in acid/base balance.

However, this process of RBC uptake of NH_3_ and export of NH_4_^+^ into the urine presumably also selectively retains protium that is returned to the circulation, while deuterium is then selectively excreted in the urine via NH_4_^+^. Essentially, through this complex process, we hypothesize that protons (^1^H) reenter the circulation and deuterium has become enriched in the aqueous medium in the loop of Henle. These allegedly deuterium-enriched fluids are finally excreted as urine.

## 12 Does glutamine supply deuterium-depleted protons to the neuronal mitochondria?

Glutamate is not only the most abundant excitatory neurotransmitter, but it is also the precursor to γ-aminobutyric acid (GABA), the predominant inhibitory neurotransmitter. Abnormal levels of extracellular glutamate impair neurotransmission and cause neurodegeneration and neuronal apoptosis. One of the most important roles of astrocytes is to assure that glutamate does not reach toxic levels in the synapse (Mangia et al., 2012).

Astrocytes also play an essential role in the brain to clear excess T-ammonia, protecting the neurons from T-ammonia toxicity (Rose et al., 2013). In achieving this goal, they also protect from glutamate neuroexcitotoxicity, because they take up glutamate that is released into the synapse and use the T-ammonia to convert the glutamate to glutamine. The so-called astrocyte-neuron glutamate-glutamine cycle prevents overstimulation of neuronal N-Methyl-D-Aspartate (NMDA)/ α-amino-3-hydroxy-5-methyl-4-isoxazolepropionic acid (AMPA) receptors, which can lead to cell death (Bröer and Brookes, 2001). The process of glutamate uptake by astrocytes, conversion to glutamine, and transport back to neurons is fast, happening in the order of seconds (Uwechue et al., 2012). A question is whether it is fast enough to predominantly avoid picking up a deuteron via exchange with protons in the side chain amide group while in transit. If so, then this whole process might be a mechanism to deliver ^1^H to the mitochondria. Note that the amide group in glutamine exchanges protons with deuterons in the aqueous medium at a much lower rate than do protons bound to oxygen in hydroxyl and carboxyl groups (Grocholska and Bąchor, 2021).

### 12.1 The glutamate/GABA-glutamine cycle

Glutamate and GABA are the principal excitatory and inhibitory neurotransmitters in the brain. They are both extensively recycled between neurons and astrocytes. Astrocytes take up these neurotransmitters from the synapse following their release by neurons. In the cytoplasm, astrocytes convert glutamate to glutamine via glutamine synthetase, and then return the glutamine to the neurons, which, in turn, resynthesize glutamate from glutamine, in their mitochondria. GABAergic neurons further metabolize glutamate via glutamic acid decarboxylase to produce GABA (Andersen, 2025). Infants with a genetically defective version of glutamine synthetase suffer from severe brain malformations, multi-organ failure and neonatal death, attesting to the importance of this enzyme to metabolism (Häberle et al., 2005).

### 12.2 Aquaporin 4 and potassium channels in astrocytes

Aquaporin 4 (AQP4) is the primary water channel in the central nervous system, and it is concentrated in the end feet of astrocytes. Curiously, AQP4 channels are localized in close proximity to inward rectifier potassium channels (Kir 4). Potassium is released from neurons into the extracellular space during repolarization of the action potential and is then rapidly taken up by astrocytes via Kir 4. Glutamate is released into the synapse during synaptic activity and is taken up by astrocytes via glutamate transporters on astrocyte processes (Xiao and Hu, 2014).

AQP4 channels import not only water but also NH_3_, because it is an uncharged molecule of similar size. Similarly, Kir 4 takes up NH_4_^+^ in addition to K+, again, because of similar size and charge properties. So, both the AQP4 channels and the Kir 4 channels can import T-ammonia.

Experiments on cultured astrocytes have shown that AQP4 is upregulated in the presence of high levels of T-ammonia (Rama Rao et al., 2003). This explains the feature of brain swelling due to the uptake of excess water by astrocytes in association with hepatic encephalopathy. But it also implies that astrocytes actively take up more NH_3_ via upregulated aquaporin channels. An experiment on cultured astrocytes exposed to ammonium chloride found that AQP4 was sharply upregulated in response to ammonium exposure, and this was mediated through phosphorylation of p38 (Pan et al., 2010). On the other hand, both Kir4.1 and Kir5.1 were *downregulated* in hyperammonemic mice, suppressing the uptake of NH_4_^+^ but also disturbing potassium homeostasis (Lichter-Konecki et al., 2008; Rangroo Thrane et al., 2013). We hypothesize that the upregulation of AQP4 and the simultaneous downregulation of Kir4.1 and Kir5.1 in response to excess T-ammonia in the vasculature is a strategy to selectively import deuterium-depleted NH_3_ rather than deuterium-rich NH_4_^+^ when T-ammonia levels are high.

### 12.3 Astrocytes clear glutamate from the synapse

Astrocytes convert glutamate to glutamine via glutamine synthetase (GS). The reaction catalyzed by GS is carried out through a two-step mechanism. In the first step, γ-glutamylphosphate is formed as an intermediate from glutamate, where phosphate is sourced from ATP. In the presence of this intermediate, NH_4_^+^ binds to the active site and, in the second step, it is deprotonated, releasing NH_3_ near the γ-glutamyl phosphate intermediate. The ammonia molecule then displaces phosphate, yielding free phosphate and glutamine (Issoglio et al., 2016). We hypothesize that any ammonium ion containing deuterium would preferentially lose the deuteron over a proton in this step, thus rarely delivering an NH_3_ molecule containing ^2^H.

Interestingly, astrocytic density is reduced in major depressive disorder (MDD). In particular, blood vessels in gray matter from the orbitofrontal area of brains of patients suffering from MDD were specifically significantly impoverished in AQP4 expression compared to normal controls (Rajkowska et al., 2013). Low levels of AQP4 could impair glutamate/glutamine cycling, and this could be a factor in symptoms of depression, by reducing the ability of neurons to maintain low deuterium in their mitochondria. Mitochondrial dysfunction in neurons is a prevalent feature of depression (Bansal and Kuhad, 2016).

Figure 3 illustrates the glutamate-glutamine exchange between neurons and astrocytes that may serve as a mechanism for glutamine to safely deliver a deuterium-depleted ammonia molecule to the mitochondria in the neuron.

**Figure 3:**
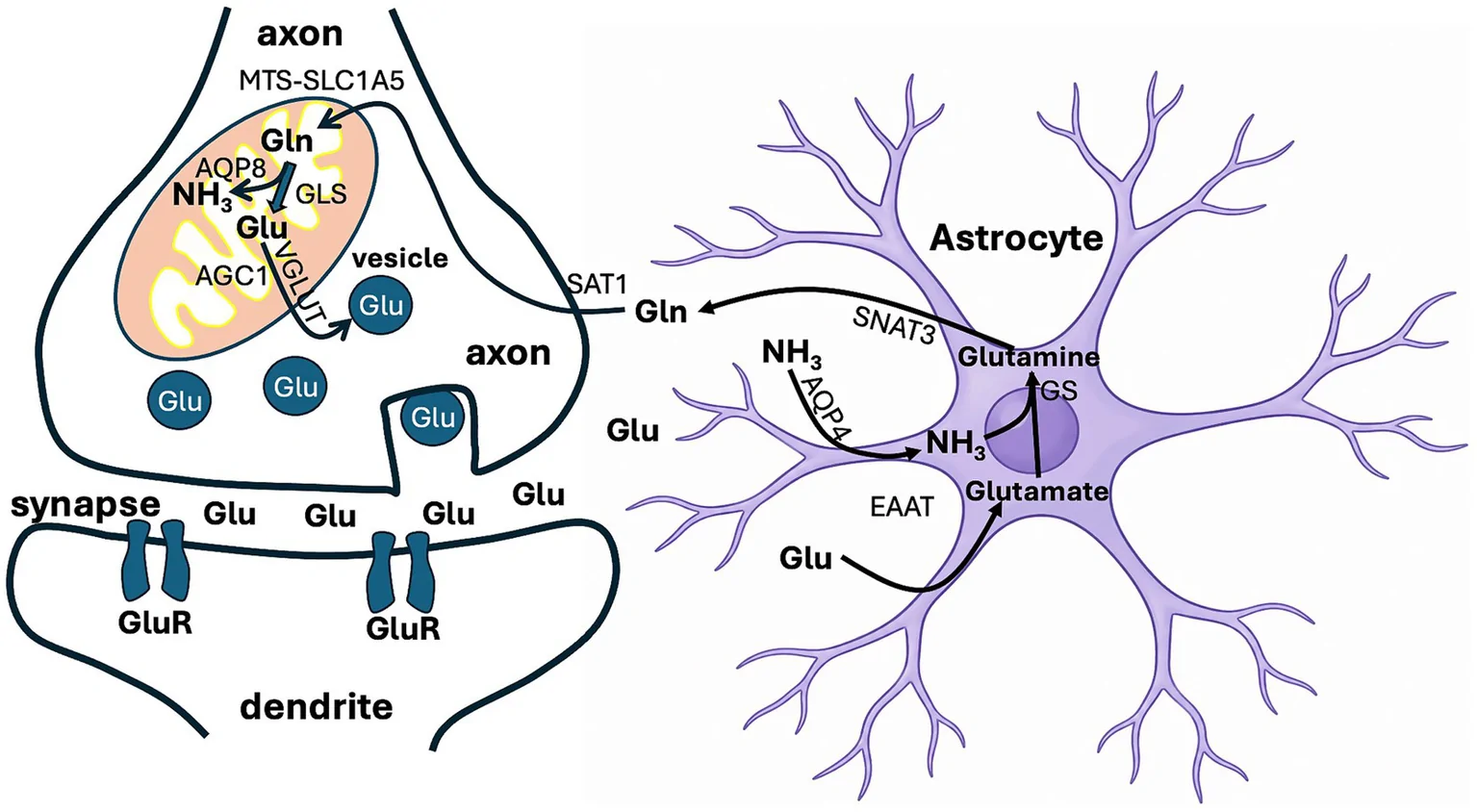
Schematic of the glutamate-glutamine cycle, whereby neurons release glutamate into the synapse from vesicles, and astrocytes take up the glutamate and convert it to glutamine, by combining it with NH_3_. The glutamate receptors in the dendrite transmit a signal by binding to glutamate, but do not take it up. The glutamine is transported back to the neuron and taken up by mitochondria, which convert it back to glutamate in the matrix, releasing NH_3_. The NH_3_ is actively imported into the intermembrane space. It is likely enriched in ^1^H relative to ^2^H. The glutamate is then transported back into the vesicles and released into the synapse for a repeat cycle. Glu: glutamate; Gln: glutamine; NH_3_: ammonia; AQP4: aquaporin-4; AQP8: aquaporin-8; GLS: glutaminase; GS: glutamine synthetase; AGC1: aspartate/glutamate carrier 1; VGLUT: vesicular glutamate transporter; EAAT: excitatory amino acid transporter; SAT1: system A amino acid transporter 1; MTS-SLC1A5: mitochondrial glutamine transporter; SNAT3: sodium-coupled neutral amino acid transporter 3.

### 12.4 T-ammonia handling in the mitochondrial matrix: supplying ^1^H to the intermembrane space?

Aquaporin-8 (AQP8) is the primary aquaporin expressed in the inner mitochondrial membrane (IMM). It plays a key role in mediating water transport into and out of the mitochondrial matrix, contributing to the organelle’s volume regulation. Because AQP8 is highly expressed in the inner membrane, water freely moves from the matrix into the intermembrane space and vice versa (Calamita et al., 2005). AQP8 is known as a “peroxiporin” because it can transport not only water but also ammonia and hydrogen peroxide across the membrane. It exclusively transports NH_3_ but not NH_4_^+^ (Saparov et al., 2007). The mitochondrial matrix has a very high pH of around 7.9, which means that∼6% of the T-ammonia is present in the gas form. The porous membrane then allows the ammonia to be siphoned off into the intermembranes space, delivering an overabundance of ^1^H to it (we hypothesize). As the ammonia is removed, more ammonia appears while dissociation equilibrium is continuously reestablished. Because the intermembrane space is much more acidic, very little of the T-ammonia remains as a gas on that side of the membrane, so ammonia naturally crosses from the matrix into the intermembrane space.

### 12.5 Liver failure and hepatic encephalopathy

Liver failure results in elevated levels of T-ammonia in the blood, because the damaged liver is unable to detoxify T-ammonia via the production of urea (Vilstrup et al., 2024). This T-ammonia readily crosses the blood–brain barrier and causes toxic effects in the brain. Increased T-ammonia concentrations in the brain significantly alter brain glutamate levels, glutamate uptake, and glutamate receptor function. In rat models, acute liver failure causes an upregulation of extracellular glutamate levels. T-ammonia is likely a major contributing factor to the brain edema and seizures associated with hepatic encephalopathy (Chan and Butterworth, 2003).

Glutamate plays a critical role in inducing brain swelling. Activation of the metabolomic glutamate receptors (mGluR) increases the rate of tissue swelling in rat hippocampal slices. During cerebral edema, excessive amounts of glutamate are released, and it causes increased expression of AQP4 (Gunnarson et al., 2008), promoting the uptake of not only water but also ammonia into astrocytes. The glutamate can then be combined with the ammonia to produce glutamine, which, in our view, is a carrier of ^1^H (protons). The removal of both the stressor ammonia and the neurotoxin glutamate from the circulation is achieved by the astrocytes through this process.

During hepatic encephalopathy, astrocytes upregulate AQP4 (Pan et al., 2010) and downregulate Kir4.1 channel expression (Lichter-Konecki et al., 2008). This causes both edema (which is primarily due to astrocyte swelling) and excess potassium in the external space, due to impaired potassium uptake by astrocytes (Obara-Michlewska et al., 2011). But it also means that T-ammonia is taken up by the astrocytes primarily as the gas rather than the dissolved cation. The fact that glutamate induces these changes in expression makes sense if one acknowledges that glutamate can be converted to glutamine both as a way to detoxify ammonia and to create a carrier molecule for ^1^H, which the astrocytes immediately deliver to adjacent neurons. In other words, might it be the case that astrocytes are able to capitalize on excess T-ammonia in the circulation to facilitate the preferential delivery of protons rather than deuterons to neurons?

## 13 Discussion

A recent scoping review paper on nutritional deuterium depletion stated in the conclusion: “To summarize the main findings, deuterium possesses profound biological effects, and deuterium depletion has major roles for example: in the prevention of cancer development with various anticancer effects, it lowers the rates of depression, reduces circulating glucose in diabetes, stimulates long-term memory, increases lifespan, and enhances sport performance” (Korchinsky et al., 2024). This leaves no doubt that deuterium matters in biology.

Deuterium homeostasis is defined as the mechanism by which the body regulates the amount of deuterium that is present in the cytoplasm, in various organelles and in the extracellular spaces (Seneff and Kyriakopoulos, 2025b; Seneff et al., 2026). The concept mostly concerns the hypothesis that the mammalian organism maintains reduced levels of deuterium in the organelles, especially the mitochondria, and concentrates deuterium in the extracellular spaces, especially in the bones, joints, fascia, and skin, as well as sequestering deuterium in carbon-nitrogen ring molecules and excreting it through the feces and urine (Seneff et al., 2025). In this paper, we have carefully examined metabolic processes that involve small hydrogen-containing gas molecules, from the perspective of deuterium homeostasis. We suggest that much of the complexity found in the metabolism of these gas molecules revolves around strategies to reduce deuterium content in mitochondrial water.

We have presented evidence here that gut microbes produce hydrogen gas that is severely reduced in deuterium, and they then utilize this gas as a reducing agent to produce small organic molecules, most notably butyrate, that fuel the mitochondria in host cells. Methane gas, derived from the molecular hydrogen, is easily converted into formate, which is an excellent source for *de novo* synthesis of CH_3_-THF, which, in turn, fuels the methylation pathway. We argue that recycling of methyl groups via TMA metabolism further scrubs deuterium from the organic molecules that support 1C metabolism.

H_2_S and other sulfur-containing gases, including DMS and CH_3_SH, also appear to play an important role in supplying ^1^H protons to the mitochondria. Gut microbes are also heavily involved in this process. H_2_S-producing bacteria are upregulated in the gut in association with Crohn’s disease (Mottawea et al., 2016). Cancer cells actively produce CH_3_SH and deliver it to tumor-resident cells, potentially as a resource to help restore their mitochondrial health (Philipp et al., 2023b).

Ammonia is another gas that can be exploited for its low-deuterium content, but this is a complicated process that involves selective transport proteins to optimize the export of deuterons via NH_4_^+^ and the retention of protons in NH_3_. Gut microbes may play an essential role through their expression of urease that converts urea produced in the liver to T-ammonia. Gut epithelial cells actively take up NH_3_, and deliver it back to the liver (Handlogten et al., 2005). Bacterial urease activity is strongly upregulated in association with gut dysbiosis and Crohn’s disease (Ni et al., 2017). The kidneys appear to preferentially retain NH_3_, the gas, and export NH_4_^+^, the cation, into the urine.

Finally, the complex interaction between neurons and astrocytes in glutamate/glutamine exchange may also be a mechanism to deliver ^1^H to neuronal mitochondria. The glutamate/glutamine cycle is dysregulated in association with various tauopathies present in neurodegenerative disease (Sidoryk-Węgrzynowicz et al., 2024).

This paper presents a strictly hypothetical account of the possible role of deuterium in the metabolism of small molecular gases. What is needed now is several carefully designed experiments to separate fact from fiction. Appropriate experiments could include controlled microbial cultures with defined isotopic compositions of water and substrates; gas chromatography coupled with isotope-ratio mass spectrometry (GC-IRMS), or other isotope-ratio methods, for H_2_, CH_4_, volatile sulfur compounds, and ammonia; isotope-resolved metabolomics for short-chain fatty acids and glutamine; gene deletions or enzyme inhibitors; and direct measurements of mitochondrial matrix water or exchangeable hydrogen under rigorously controlled conditions, to name a few.

### 13.1 Limitations

The biggest limitation of this work is the weak foundation supporting the idea that the molecular hydrogen gas produced by the gut microbes fractionates out most of the deuterium from the gas. It is primarily based on a 1961 paper (Krichevsky et al., 1961) showing that a marine microbe produces hydrogen gas that is 80% depleted in deuterium from organic sources such as glucose and formate. I was unable to find any more recent papers that had repeated this experiment for other hydrogenase enzymes. However, support also comes from research showing that a microbial hydrogenase has a very high deuterium KIE (reaching as high as 43 in an acidic medium) (Greene et al., 2015).

Another limitation is the strength of the evidence supporting the claim that mitochondrial ATP synthase nanomotors are highly sensitive to deuterium. It is plausible, based on the distinct physical properties of protons and deuterons, that a deuteron binding to the Asp61 residue in the C protein slows down the nanomotors, creating a stutter (Aksimentiev et al., 2004). The degree to which this causes damage, and the degree to which the cell filters deuterium out of mitochondrial water, remain as unknown variables. It would be very challenging to design an experiment that would measure the deuterium level in mitochondrial water, and this remains problematic.

A third limitation is the generalization from molecular hydrogen to other small molecular gases containing hydrogen. There is limited evidence to support this idea from measures of deuterium content of methane in volcanic gases (Ricci et al., 2024), and from the evaporation of water from raindrops as they fall to the ground (Peng et al., 2007; Chen et al., 2021). The fractionation that occurs when water evaporates from a raindrop does not easily generalize to evaporation of other hydrogen-containing volatile molecules.

A fourth limitation concerns the H/D experiments that are the basis for the idea that certain carbon-nitrogen-containing rings can permanently trap and sequester deuterium, and that the organism makes use of this feature to help sequester deuterium away from the mitochondria. The experiments where such trapping has been achieved are all based on immersing the molecule in water that is highly enriched in deuterium, an unrealistic scenario for living organisms.

A final limitation is insufficient evidence that gases in equilibrium with dissolved ionic forms tend to fractionate out deuterium in the gas. In aqueous biological systems, species such as H₂S/HS^−^, NH₃/NH₄^+^, and TMA/TMAH^+^ exist in equilibria that depend on pH and their respective pKa values. Exchangeable hydrogen atoms may undergo complex isotope-exchange processes. Current evidence is insufficient to support selective separation of protium and deuterium in these systems.

In some sense, this paper has approached the problem backwards: we ask the question, does the way the human organism deals with small molecular gases containing hydrogen make sense if a primary goal is to enrich the content of the mitochondria with ^1^H relative to ^2^H? It seems that the evidence from known biochemical pathways supports this view. And it also brings out the essential nature of the gut microbiome in achieving such a goal. However, the proposed mechanisms remain hypothetical and unverified.

## 14 Conclusion

In this paper, we have presented a hypothesis that small hydrogen-containing gases may have a special and unique role to play in deuterium homeostasis, due in part to the idea that deuterium tends to stay behind in the aqueous medium. It appears that many of the processes that take place in the human organism that involve these gases are logically designed to take advantage of the low deuterium content. The gut microbes may play a pivotal and essential role in providing the host with nutrients derived from these gases that are expected to also be low in deuterium. Research on deuterium is still in its infancy, and many more studies need to be done by experts in biochemistry and physics to either validate or disprove these ideas.

We hope that this paper will inspire others to conduct research to clarify the role of deuterium in human metabolism and the processes by which mitochondria are protected from deuterium overload. If our ideas around the role of small molecular gases, many of which are produced by the gut microbes, in deuterium homeostasis can be proven, it would greatly alter our interpretation of the mechanisms of disease processes.

## Data Availability

The original contributions presented in the study are included in the article/supplementary material, further inquiries can be directed to the corresponding author.
